# VIMO: A Visual-Inertial-Magnetic Navigation System Based on Non-Linear Optimization

**DOI:** 10.3390/s20164386

**Published:** 2020-08-06

**Authors:** Jingzhe Wang, Leilei Li, Huan Yu, Xunya Gui, Zucheng Li

**Affiliations:** School of Automation, Beijing Institute of Technology, Beijing 100081, China; weskerredfield@aliyun.com (J.W.); yuhuan.bit@gmail.com (H.Y.); guixunya@hotmail.com (X.G.); lizucheng66@sina.com (Z.L.)

**Keywords:** visual-inertial navigation, yaw estimation, magnetic information, non-linear optimization

## Abstract

Visual-inertial navigation systems are credited with superiority over both pure visual approaches and filtering ones. In spite of the high precision many state-of-the-art schemes have attained, yaw remains unobservable in those systems all the same. More accurate yaw estimation not only means more accurate attitude calculation but also leads to better position estimation. This paper presents a novel scheme that combines visual and inertial measurements as well as magnetic information for suppressing deviation in yaw. A novel method for initializing visual-inertial-magnetic odometers, which recovers the directions of magnetic north and gravity, the visual scalar factor, inertial measurement unit (IMU) biases etc., has been conceived, implemented, and validated. Based on non-linear optimization, a magnetometer cost function is incorporated into the overall optimization objective function as a yawing constraint among others. We have done extensive research and collected several datasets recorded in large-scale outdoor environments to certify the proposed system’s viability, robustness, and performance. Cogent experiments and quantitative comparisons corroborate the merits of the proposed scheme and the desired effect of the involvement of magnetic information on the overall performance.

## 1. Introduction

As befits one of the most crucial problems in robotics, SLAM has received much attention for the past two decades and opened up many new vistas for autonomous robots, alongside numerous proposed approaches and schemes for implementing it, which fall into three categories by sensing modality: Laser SLAM, visual SLAM, and visual-inertial SLAM. Laser SLAM preponderated in the early stages of the development of SLAM by virtue of its high precision, long range, and capacity for obstacle avoidance. Notwithstanding its advantages, its scale in size and weight means its application is confined to platforms allowing a limited load and a capacity enough for carrying a laser scanner, which therefore circumscribes the application range and agility of the platform. Being cost-effective, lightweight, and efficient in energy consumption, cameras, or vision-based sensors in general, are in the ascendant, which is also attributed to its informative representation of geometry in a single image caught on camera. A desirable consequence of images being abundant in information is the capacity to retrieve previously registered scenes, known as scene recognition, so as to perform loop closure to curb drift in estimates.

For a vision-based SLAM system or those reliant mainly on visual perception, four components are deemed indispensable, including visual tracking, filtering/optimization, loop closure, and mapping, each of which holds systemic effects on the overall performance of a system and to which various solutions have been conceived, implemented, and experimented. Approaches to tracking visual cues can be categorized into such two classes as feature-based and direct methods according to how they implement data association. Feature-based methods rely on the descriptors of features extracted in images to associate image points appearing across several images, and points existing in more than one images can then be used to estimate poses with stability due to the invariance of descriptors. Apart from the relatively high computational cost of extracting features and descriptors, one of the penalties to feature-based methods is their lack of resistance to texture-less views. The descriptor, dependent on features being identifiable, does not contain the amount of information enough to perform data association. Direct methods, however, eschew this problem by direct exploitation of changes in illumination and by minimization of photometric errors rather than geometric ones, thereby being more impervious to scarcity of texture as well as driving down computational consumption since they don’t have to describe features. The concept behind every formulations of estimation is probabilistic modelling with noisy measurements as input to estimate certain parameters. Typical models are based on maximum a posterior (MAP) in which the final estimates maximize the a posteriori probability given existent measurements. For pure visual SLAM, the probabilistic model is usually based on Maximum Likelihood (ML) in the absence of prediction models. Earlier SLAM schemes estimate states mainly in a filtering way in which the prior parameters are always marginalized out to focus merely on current states for containment of computation, i.e., all the previous states are fixed, which is bound to affect precision. Recently nonlinear optimization-based methodology has begun to gain ground among scholars alongside increases in the performance of hardware and decreases in its cost. Due to the sparsity inherent in the Hessian matrix of BA (Bundle Adjustment), the computational complexity of estimation in SLAM is lighter than would be conceived. By benefiting from the sparsity and properly assigning tasks among several parallels and with existent optimization modules including Ceres, g2o, iSAM, and gtsam which have been leveraged across many platforms of navigation, optimization-based systems can run smoothly within reasonable time but still need to marginalize out older states to restrict the number of keyframes. The factor graph based on fast incremental matrix factorization, on the other hand, allows for a more accurate and efficient solution by virtue of recalculating only the matrix entries in need of change, whereby the system can considerably slash computation while holding the whole trajectory.

The inertial measurement unit (IMU), light in weight, size, and price, and often in the form of a MEMS (Micro-Electro-Mechanical System), has been gaining in popularity as a source of inertial information complementary to vision since it recovers poses from motion itself and permits rendering observable the metric scale of monocular vision and the direction of gravity whereas visual constraints, in reverse, given accurate data association, can check error accumulation in the integration of IMU measurements (angular velocity, acceleration). The fusion of visual and inertial information began with loosely-coupled mechanisms and has now transitioned into tightly-coupled ones. The procedure of propagating and predicting states with inertial measurements between two consecutive frames and later using a visual image (or several) as an observation to update estimates is characteristic of loosely-coupled mechanisms which are straightforward but fall short of gratifying decision because they fail to take into account the correlation between the two types of data, as opposed to which, tightly-coupled ones fare better by incorporating variables specific to vision (the coordinates of landmarks) and those to inertia (gyroscope and accelerometer biases) into the whole set of optimization states, which in essence utilizes the complementary attribute to a greater extent. To ameliorate the additional computational expense incurred by the introduction of inertial constraints into the optimization graph, in which case the Hessian matrix is no longer as sparse as that of purely visual models, pre-integration on manifold may be adopted to reduce calculation for optimization and bias correction.

Merging inertial and visual information leads to the angles of pitch and roll being observable with the last dimension of attitude, namely yaw, still unobservable and bound to drift over time. It is conceivable that high accuracy in yawing estimation can bolster the overall precision as every pose is estimated partly based on previous estimates and is therefore affected by their yaw angles. The magnetometer has long been employed in the field of navigation, often in combination with other types of apparatus such as an IMU. The magnetometer, as its name reveals, is a type of sensors that measure magnetic density in a magnetic field, especially the Earth’s magnetic field (EMF), one of the Geophysical Fields of the Earth (GFE) including the Earth’s Gravitational Field (EGF). It has been used in a wide range of commercial and military applications, mostly for directional information [1], whereas another way of using it for motion estimation is through measuring magnetic field gradients so as to acquire velocity information as in [2] which formulated a sensing suite consisting of a vision sensor and a MIMU (Magneto-Inertial Measurement Unit) that, on the presupposition of stationary and non-uniform magnetic field surroundings (particularly indoor environments), can render the body speed observable. The said two manners of employing the magnetometer imply that for this sensor there is a dichotomy in how to process its readings between outdoor and indoor scenarios, and it will not be straightforward to reconcile them.

However, little research has been done about integrating visual-inertial frameworks with magnetic observation that could ably suppress the cumulative azimuth error and further facilitate navigation. It seems that the magnetometer is not so much appealing for scholars in the area of SLAM as it ought to be. What keeps magnetometers from being adopted might be its liability to magnetic disturbance. Ubiquitous sources magnetic interference from ferrous metals and even from the platform itself would entail the system both growing in size and demanding more intricate handling. Since applications of SLAM are always towards compactness and generality, the magnetometer has remained out of favour.

We gather that despite the magnetometer’s weaknesses, outdoor environments might still be in accord with its characteristics with appropriate measures taken, and therein lies the main motivation of the paper.

In this paper, we present a visual-inertial-magnetic system of navigation based on graph optimization that, apart from visual and inertial measurements, utilizes the geomagnetic field to restrain drift in yaw. From devising the scheme to verifying its superiority, the following tasks have been followed through along the way:

1. Exhaustive mathematic deductions have been made to support the proposed system theoretically and mathematically, embodying observation models, least squares problems concerned with initialization, the novel optimization framework that fuses visual-inertial-magnetic information, and a novel way of loop-closure formulation aimed at enhancing the robustness to false loops.
2. A complete and reliable procedure of initialization for visual-inertial-magnetic navigation systems is presented.
3. An effective and efficient optimization using visual-inertial-magnetic measurements as observation is established.
4. A suite of sensors and a CPU with other hardware, capable of data acquisition and real-time operation, has been assembled.
5. The system has been tried and tested on several datasets collected in large-scale outdoor environments. Analysis and comparison of the experiment results attest to the feasibility, efficacy, and excellence of the proposed system.

## 2. Related Work

Since [3] came out, large numbers of well-designed systems [4,5,6,7,8] have swollen the ranks of SLAM schemes. Copious studies have been done on visual-inertial SLAM along with various applications that have since been on a continuum to full maturity [9,10,11]. Until today, scholars are still endeavoring to bring it to perfection with novel ideas and approaches [12,13,14,15,16]. Incipient methods fuse inertial and visual measurements loosely where the two types of information are processed, filtered, and used for estimation all separately [17]. In overcoming the notorious inconsistency of loosely-coupled approaches, researchers have come to appreciate that the advantages of fusing tightly, such as improved consistency [18,19]. Whether sources of information are amalgamated loosely or tightly, filtering mechanisms hold more likelihood to arrive at a suboptimal solution in the wake of linearizing all previous states. In break with traditional filter-based methods, non-linear optimization employed in SLAM has been deemed more desirable with its high precision and tolerable computational requirements. Reference [20] copes with holding on to and optimizing an entire trajectory by what the authors call ‘full smoothing’, but the ever increasing complexity in line with the incremental map and trajectory largely limits its applicability. Reference [21] is generally recognized as one of most early mature systems using non-linear optimization based on sliding window and marginalization for containment of problem size. To make a virtue of escalated computation due to the addition of inertial measurements, reference [22] proposes what is called the IMU preintegration technique that integrates on manifold a segment of measurements between two time points with the Jacobian matrices maintained for correction. In that way, changes in linearization will not need complete re-integration but a few minor adjustments with respect to the Jacobian matrices as long as they are not too large.

Another property of visual-inertial SLAM that comes to scholar’s attention is its initialization and what its does is to work out several parameters describing the system’s initial states and sensors’ relation. Sfm calls for enough motion to be reliable while estimation of gravity’s direction is better off under stationary condition. This contradiction suggests that visual-inertial initialization can not be trifled with. Reference [23] proposes a deterministic closed-form method that can recover gravity’s direction and scalar factor, but fails to take cognizance of IMU biases, making the system less stable than it would otherwise be. Reference [17] estimates not only IMU biases but velocity using an EFK but the convergence takes long. Reference [24] initializes system on the assumption that MAVs take off flat with as small inclination as possible, which is, of necessity, unlikely to be the case in practice. Reference [25], much like [24], relies on alignment with gravity at the beginning to initialize. In [26,27], initialization does not calculate gyroscope biases, derogating from the precision.

As it develops, SLAM has begun to go beyond passively observing surroundings to actively exploring environments so as to gain coverage, hence the name ‘active SLAM’ [28]. So-called active SLAM integrates SLAM itself with path planing. Using this technique, a system covers an area autonomously while performing plain SLAM.

Reference [29] is aimed at solving active SLAM problems where coverage is required and certain constraints are imposed. With that end in view, reference [29] proposes a solution to it that focuses on minimization and area coverage within an MPC (Model Predictive Control) framework. It uses a sub-map joining method to improve both effectiveness and efficiency. The D-opt MPC problem is resolved with recourse to a graph topology and convex optimization and the SQP method is employed to address the coverage problem.The main contribution of [29] is it presents a new method capable of generating a sound collision-free trajectory so as to better perform coverage tasks than many other systems do.

Reference [30] presents an effective indoor navigation system for the Fetch robot. The main idea is founded on sub-mapping and DeepLCD and the system is implemented using Cartographer and AMCL (adaptive Monte Carlo localization). The main method comprises mapping and on-line localization modules and sequential sub-maps are generated by fusing data from a 2D laser scanner and a RGBD camera. AMCL is used to perform accurate localization according to image matching results. Not only the localization system itself but novel evaluation methods for it are presented in the paper and by using it the robustness and accuracy of the system is demonstrated.

Reference [31] presents a navigation method whereby a flying robot is able to explore and map an underground mine without collision. Simulations have verified that the system performs as well as the authors expected whether the robot is circling above flat or sloping ground. The authors also claim in the paper that the system is as simple as it is reliable.

## 3. Preliminaries

### 3.1. Notation

We employ the following symbols throughout this paper. ${\left(.\right)}_{W}$ denotes the world frame, ${\left(.\right)}_{B_{k}}$ the *k*th body frame which doubles as the *k*th IMU frame as aligned with the body frame, ${\left(.\right)}_{C_{k}}$ the *k*th camera frame, and ${\left(.\right)}_{M_{k}}$ the *k*th magnetometer frame. As for measurements, $w_{k}$ represents the gyroscope’s readings, $a_{k}$ the accelerometer’s, and $h_{k}$ the magnetometer’s, at *k*th frame. ${\left(u,v\right)}_{k}$ the coordinates of a feature point in the *k*th image.

The overall states to be estimated are expressed in such a vector as $x={\left[\begin{array}{c} P,q,V,bg,ba,q_{BC} \end{array}\right]}_{B_{k}}$ with *P* the position, *q* the body orientation quaternion, *V* the velocity, $b_{g}$ and $b_{a}$ the gyroscope’s and accelerometer’s biases respectively at the *k*th frame, $q_{BC}$ the transformation from frame *C* to frame *B* intrinsic to the installation and thus considered to be invariant. $L_{W}$ symbolizes a landmark expressed homogeneously with $L_{W}=\left[\begin{array}{c} x,y,z,1 \end{array}\right]$.

$\theta^{\times }$ represents the askew matrix of the vector θ:

(1) $$\begin{array}{c} \theta^{\times }=\left[\begin{array}{c} \theta_{1}\\ \theta_{2}\\ \theta_{3} \end{array}\right]=\left[\begin{array}{ccc} 0 & -\theta_{3} & \theta_{2}\\ \theta_{3} & 0 & -\theta_{1}\\ -\theta_{2} & \theta_{1} & 0 \end{array}\right] \end{array}$$

Note that ${\theta^{\times }}^{T}=-\theta^{\times }$ by its very definition.

### 3.2. Useful Properties of SO(3)

SO(3), the special orthogonal group in 3 dimensions, describes rotations in 3D space, with its corresponding vector so(3) in parameter. SO(3) and so(3) are related to each other through the exponential and logarithmic maps:

(2) $$R\left(\theta \right)=\mathrm{Exp}\left(\theta \right)=I+\frac{sin||\theta ||}{\left||\theta |\right|}\theta^{\times }+\frac{1-cos||\theta ||}{{\left||\theta |\right|}^{2}}{\theta^{\times }}^{2}$$

(3) $$\theta =\mathrm{Log}\left(R\left(\theta \right)\right)=\frac{\left||\theta |\right|}{2sin||\theta ||}{\left(R-R^{T}\right)}^{\vee }$$

(4) $$\left||\theta ||=arccos(\frac{trace(R)-1}{2}\right)$$

Four properties of SO(3) are essential in IMU pre-integration and Jacobian computation for optimization and thus merit a mention:

- commutative
  (5) $$\theta^{\times }\phi =-\phi^{\times }\theta$$
- approximation
  (6) $$Exp\left(\theta \right)\approx I+\theta^{\times }$$
- adjoint
  $$\begin{array}{cc} R\cdot Exp(\theta ) & =Exp(Adj_{\left(R\right)}\cdot \theta )\cdot R \end{array}$$
  (7) $$\begin{array}{cc} \; & =Exp(R\cdot \theta )\cdot R \end{array}$$
  $$\begin{array}{cc} Exp(\theta )\cdot R & =R\cdot Exp(Adj_{\left(R\right)}^{T}\cdot \theta ) \end{array}$$
  (8) $$\begin{array}{ccc} \; & & =R\cdot Exp(R^{T}\cdot \theta ) \end{array}$$
- the righ-hand jacobian
  (9) $$\begin{array}{cc} Exp(\theta +\delta \theta ) & \approx Exp\left(\delta \right)Exp(J_{r}\left(\theta \right)\delta \theta ) \end{array}$$
  (10) $$\begin{array}{cc} Exp(\theta )Exp(\theta +\delta \theta ) & \approx Exp(\theta +J_{r}^{-1}\left(\theta \right)\delta ) \end{array}$$
  (11) $$\begin{array}{cc} Log(Exp(\theta )Exp(\theta +\delta \theta )) & \approx \theta +J_{r}^{-1}\left(\theta \right) \end{array}$$
  (12) $$\begin{array}{cc} J_{r}\left(\theta \right) & =I-\frac{1-cos||\theta ||}{{\left||\theta |\right|}^{2}}{\left[\theta \right]}^{\times }+\frac{\left||\theta -sin||\theta |\right|}{{\left||\theta |\right|}^{3}}{{\left[\theta \right]}^{\times }}^{2} \end{array}$$
  (13) $$\begin{array}{cc} J_{r}^{-1}\left(\theta \right) & =I+\frac{1}{2}{\left[\theta \right]}^{2}-\left(\frac{1}{{\left||\theta |\right|}^{2}}-\frac{1+cos||\theta ||}{2||\theta ||sin||\theta ||}\right){{\left[\theta \right]}^{\times }}^{2} \end{array}$$

Note that there is also $J_{l}$ called the left-hand jacobian as opposed to $J_{r}$. We shall refer to them as $J_{r}$ and $J_{l}$ for the following content.

## 4. VIMO Measurement Models

### 4.1. Vision

We use a pinhole camera as a source of visual measurement. Under the pinhole camera projection model, a 3D point is projected onto a plane with Z normalized and then projected onto the image plane with projection parameters intrinsic to the camera:

(14) $$\left[\begin{array}{c} u\\ v\\ 1 \end{array}\right]=\frac{1}{Z}\left[\begin{array}{ccc} f_{x} & 0 & c_{x}\\ 0 & f_{y} & c_{y}\\ 0 & 0 & 1 \end{array}\right]\left[\begin{array}{c} X\\ Y\\ Z \end{array}\right]$$

where *u*, *v* denote the coordinates of the point projected onto the plane. In most cases, coordinates go through radial and tangential distortion on the normalized plane with parameters called distortion coefficients before scaled and displaced to form the final pixel coordinates. The distortion coefficients vary in value and number according to the lenses of cameras. Lenses with a great degree of distortion should be treated with up to 3 coefficients to express the distortion properly. The lens of the camera used in the proposed scheme is an ordinary one and only two distortion coefficients are adopted.

(15) $$\begin{array}{c} x_{dis}=x+2p_{1}xy+p_{2}\left(r^{2}+2x^{2}\right)\\ y_{dis}=y+p_{1}\left(r^{2}+2y_{2}\right)+2p_{2}xy \end{array}$$

The Jacobian matrices of pixel coordinates with respect to distorted ones, distorted ones to normalized ones, and normalized ones to 3D coordinates are present as follows:

(16) $$J_{img}=\left[\begin{array}{cc} f_{x} & 0\\ 0 & f_{y} \end{array}\right]$$

(17) $$J_{dis}=\left[\begin{array}{cc} 1+k_{1}r^{2}+k_{2}r^{4}+2k_{1}k_{2}u^{2}+4k_{2}u^{2}r^{2}+2p_{1}v+6p_{2}u\; & 2k_{1}uv+4k_{2}r^{2}uv+2p_{1}u+2p_{2}v\\ 2k_{1}uv+4k_{2}r^{2}uv+2p_{1}u+2p_{2}v\; & 1+k_{1}r^{2}+k_{2}r^{4}+2k_{1}u^{2}+4k_{2}r^{2}u^{2}+6p_{1}v+2p_{2}u \end{array}\right]$$

(18) $$J_{nor}=\left[\begin{array}{ccc} 1/Z & 0 & -X/Z^{2}\\ 0 & 1/Z & -Y/Z^{2} \end{array}\right]$$

The overall Jacobian matrix can then be obtained by applying the chain rule:

(19) $$J_{pro_{2D\to 3D}}=J_{img}\cdot J_{dis}\cdot J_{nor}$$

### 4.2. Inertia

Inertial data are obtain from an IMU at successive time instants at a frequency of 200 Hz:

(20) $$\begin{array}{cc} w_{m} & =w_{t}+b_{w}+n_{w} \end{array}$$

(21) $$\begin{array}{cc} a_{m} & =R_{BW}\left(a_{t}+g_{W}\right)+b_{a}+n_{a} \end{array}$$

where $w_{m}$ and $a_{m}$ are acceleration and angular readings, $n_{w}$ and $n_{a}$ conceived of as Gaussian white noise, $b_{w}$ and $b_{a}$ modelled as random walk, and $w_{t}$ and $a_{t}$ the angular rate and acceleration in the world frame.

The error-state kinematics in continuous time are

(22) $$\begin{array}{cc} \delta \dot{p} & =\delta v \end{array}$$

(23) $$\begin{array}{cc} \delta \dot{v} & =-R{\left[a_{m}-b_{a}\right]}^{\times }\delta \theta -R\delta a_{b}+\delta g-Rw_{n} \end{array}$$

(24) $$\begin{array}{cc} \delta \dot{\theta } & =-{\left[w_{m}-w_{b}\right]}^{\times }\delta \theta -\delta b_{w}-n_{w} \end{array}$$

(25) $$\begin{array}{cc} \delta \dot{b_{a}} & =n_{a} \end{array}$$

(26) $$\begin{array}{cc} \delta \dot{b_{g}} & =n_{w} \end{array}$$

In traditional navigation schemes where filters are often used, states are predicted by integration of inertial measurements based on the prior states, indicating that accurate estimates of the initial states are crucial since even slightly skewed gravity direction would translate into enormously egregious errors in position and velocity estimates, giving rise to complete divergence. For positioning systems based on state optimization applied in more generic environments, not only is the unknown initial orientation problematic but the necessity of the system being re-linearized after each optimization step acts as a further drag. With a view to tackling this issue, the concept of IMU pre-integration has emerged and been well applied in visual-inertial navigation. Through multiplying both sides of the kinematics by a rotation matrix intended to transform the reference frame from the world frame *W* to the beginning frame $B_{k}$ in question, relative integration terms independent of state variables and gravity can be separated from other terms, obviating the need for re-propagation.

We reckon that mid-point integration is accurate enough as well as being efficient in computation. As IMU readings arrive at regular intervals, the integration goes on step by step as follows:

(27) $$\begin{array}{cc} \Delta R_{k+1} & =\Delta R_{k}\cdot Exp\left(\left(\frac{w_{k}+w_{k+1}}{2}-b_{w}\right)\cdot \Delta t\right) \end{array}$$

(28) $$\begin{array}{cc} \Delta V_{k+1} & =\Delta V_{k}+\frac{\Delta R_{k}+\Delta R_{k+1}}{2}\cdot \left(\frac{a_{k}+a_{k+1}}{2}-b_{a}\right)\Delta t \end{array}$$

(29) $$\begin{array}{cc} \Delta P_{k+1} & =\Delta P_{k}+\Delta V_{k}\Delta t+\frac{1}{2}\cdot \frac{\Delta R_{k}+\Delta R_{k+1}}{2}\left(\frac{a_{k}+a_{k+1}}{2}-b_{a}\right)\Delta t^{2} \end{array}$$

where ΔR, ΔV, and ΔP are the pre-integration terms with the subscript ${\left(.\right)}_{k}$ denoting IMU frames. Note that the pre-integration terms are independent of state variables except biases. No re-propagation will be required after the position $P_{k}$, the velocity $V_{k}$, and the rotation $R_{k}$ change and for small alterations in biases the pre-integration will be adjusted according to the jacobians, caculated iteratively alongside pre-integration, of errors in ΔR, ΔV, ΔR with respect to the biases $b_{w}$ and $b_{a}$.

For error propagation, the error-state kinematics differ from those corresponding to Euler integration since they involve measurements at both the previous and the next time instant. The error updates are as follows:

(30) $$\begin{array}{cc} \delta \theta_{k+1}\leftarrow & \delta \theta_{k}-R_{k}J_{L}\left({\bar{w}}_{m}\Delta t\right)\delta b_{w}+R_{k}J_{L}\left({\bar{w}}_{m}\Delta t\right)n_{w} \end{array}$$

(31) $$\begin{array}{cc} \delta V_{k+1}\leftarrow & \delta V_{k}-{\left(\bar{R}{\bar{a}}_{m}\Delta t\right)}^{\times }\delta \theta_{k}+\frac{1}{2}\Delta t^{2}R_{k+1}{\bar{a}}_{m}^{\times }J_{R}\left({\bar{w}}_{m}\Delta t\right)\delta b_{w}-\bar{R}\Delta t\delta b_{a}\\ \; & -\frac{1}{2}\Delta t^{2}R_{k+1}{\bar{a}}_{m}^{\times }J_{R}\left({\bar{w}}_{m}\Delta t\right)n_{w}+\bar{R}\Delta tn_{a} \end{array}$$

(32) $$\begin{array}{cc} \delta P_{k+1}\leftarrow & \delta P_{k}+\Delta t\delta V_{k}-\frac{1}{2}{\left(\bar{R}{\bar{a}}_{m}\Delta t^{2}\right)}^{\times }\delta \theta_{k}+\frac{1}{4}\Delta t^{3}R_{k+1}{\bar{a}}_{m}^{\times }J_{R}\left({\bar{w}}_{m}\Delta t\right)\delta b_{w}-\frac{1}{2}\bar{R}\Delta t^{2}\delta b_{a}\\ \; & -\frac{1}{4}\Delta t^{3}R_{k+1}{\bar{a}}_{m}^{\times }J_{R}\left({\bar{w}}_{m}\Delta t\right)n_{w}+\frac{1}{2}\bar{R}\Delta t^{2}n_{a} \end{array}$$

(33) $$\begin{array}{cc} \delta {\mathrm{bw}}_{k+1}\leftarrow & \delta {\mathrm{bw}}_{k}+n_{\mathrm{bw}}\Delta t \end{array}$$

(34) $$\begin{array}{cc} \delta {\mathrm{ba}}_{k+1}\leftarrow & \delta {\mathrm{ba}}_{k}+n_{\mathrm{ba}}\Delta t \end{array}$$

where δ(.) indicates error states, Δt the discrete time interval between $t_{k}$ and $t_{k+1}$. Note that in the above equations ${\bar{w}}_{m}$ and ${\bar{a}}_{m}$ take the place of $\frac{w_{k}+w_{k+1}}{2}-b_{w}$ and $\frac{a_{k}+a_{k+1}}{2}-b_{a}$ and $\bar{R}$ of $\frac{R_{k}+R_{k+1}}{2}$ for notation simplicity. For the Equation (30) of rotation error propagation, the angular error is defined on the right side, i.e., $R_{true}=\delta RR$, probably opposite to most other definitions.

The jacobians $\frac{\delta \theta }{\delta b_{w}}$, $\frac{\delta V}{\delta b_{w}}$, $\frac{\delta V}{\delta b_{a}}$, $\frac{\delta P}{\delta b_{w}}$, $\frac{\delta P}{\delta b_{a}}$ calculated by (30), (31), and (32) are used for correction of pre-integration values in response to variations in biases. If the norm of the bias vector ${\left[\begin{array}{cc} b_{w} & b_{a} \end{array}\right]}^{T}$ (rad/s${}^{2}$ for $b_{w}$ and m/s${}^{2}$ for $b_{a}$) reaches above a threshold of $10^{-4}$, re-propagation over this period is warranted and executed as the linearization point has changed too much.

According to (30)–(34), the error-state transition matrix is

(35) $$F_{\delta x}=\left[\begin{array}{ccccc} I & 0 & 0 & -R_{k}J_{L}\left({\bar{w}}_{m}\Delta t\right) & 0\\ -{\left(\bar{R}{\bar{a}}_{m}\Delta t\right)}^{\times } & 0 & \frac{1}{2}\Delta t^{2}R_{k+1}{\bar{a}}_{m}^{\times }J_{R}\left({\bar{w}}_{m}\Delta t\right) & -\bar{R}\Delta t\\ -\frac{1}{2}{\left(\bar{R}{\bar{a}}_{m}\Delta t^{2}\right)}^{\times } & \Delta t & I & \frac{1}{4}\Delta t^{3}R_{k+1}{\bar{a}}_{m}^{\times }J_{R}\left({\bar{w}}_{m}\Delta t\right) & -\frac{1}{2}\bar{R}\Delta t^{2}\\ 0 & 0 & 0 & I & 0\\ 0 & 0 & 0 & 0 & I \end{array}\right],$$

and the Jabocian matrix of pre-integration with respect to the noise vector.

(36) $$F_{i}=\left[\begin{array}{cccc} R_{k}J_{L}\left({\bar{w}}_{m}\Delta t\right) & 0 & 0 & 0\\ -\frac{1}{2}\Delta t^{2}R_{k+1}{\bar{a}}_{m}^{\times }J_{R}\left({\bar{w}}_{m}\Delta t\right) & \bar{R}\Delta t & 0 & 0\\ -\frac{1}{4}\Delta t^{3}R_{k+1}{\bar{a}}_{m}^{\times }J_{R}\left({\bar{w}}_{m}\Delta t\right) & \frac{1}{2}\bar{R}\Delta t^{2} & 0 & 0\\ 0 & 0 & \Delta t & 0\\ 0 & 0 & 0 & \Delta t \end{array}\right]$$

The propagation of errors and covariances for pre-integration is summarized as

(37) $$\begin{array}{cc} \delta x_{k+1} & \leftarrow F_{x}\left(x_{k},{\bar{w}}_{m}\Delta t,{\bar{a}}_{m}\Delta t\right)\delta x_{k} \end{array}$$

(38) $$\begin{array}{cc} P_{k+1} & \leftarrow F_{x}P_{k}{F_{x}}^{T}+F_{i}Q_{i}{F_{i}}^{T} \end{array}$$

where $Q_{i}$ is the covariance matrix of Gaussian white noise determined from the IMU datasheet or through calibration experiments. $P_{B_{k}B_{k+1}}$ propagated by (38) during the pre-integration from $f_{B_{k}}$ to $f_{B_{k+1}}$ accounts for uncertainty, or observation noise covariances, and is used to weight inertial residuals.

### 4.3. Magnetism

Besides vision and inertia, geomagnetism is also involved to relieve localization of the accumulative yawing error that would otherwise be ever mounting up.

**Figure 1:**
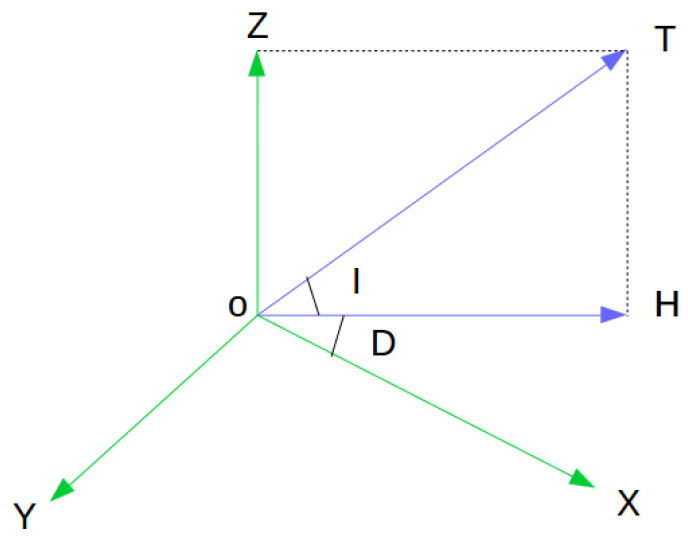
The geomagnetic and geographic coordinate frames.

As with the accelerator that can measure the gravitational field on a static platform, the magnetometer purveys readings of the projection of its surrounding magnetic field onto its body frame. While what it gauges is not the projection purely of the EMF, traditionally for centuries it has been used for bearing information. Figure 1 illustrates the vector of the total density EMF *T* and its projections on the geomagnetic and geographic coordinate axes.

In Figure 1:

- T – the vector of the total density EMF;
- X, Y, and Z – the Geographic System Coordinates;
- I – the angle of magnetic inclination;
- D – the angle of magnetic declination;

Such parameters as T, I, D can be determined by referencing geomagnetic maps that describe the geomagnetic features of various locations around the globe.

The average magnetometer’s calibration model is

(39) $$\begin{array}{c} \left[\begin{array}{c} {M_{x}}_{t}\\ {M_{y}}_{t}\\ {M_{z}}_{t} \end{array}\right]=\left[\begin{array}{ccc} k_{xx} & k_{yx} & k_{zx}\\ k_{yy} & k_{xy} & k_{zy}\\ k_{zz} & k_{xz} & k_{yz} \end{array}\right]\left[\begin{array}{c} M_{x}\\ M_{y}\\ M_{z} \end{array}\right]-\left[\begin{array}{c} b_{m_{x}}\\ b_{m_{y}}\\ b_{m_{z}} \end{array}\right] \end{array}$$

where: ${\left[\begin{array}{ccc} M_{x} & M_{y} & M_{z} \end{array}\right]}^{T}$ is the magnetometer’s raw readings that are a coefficient matrix and a bias vector away from the true magnetic projection on the sensor’s body frame ${\left[\begin{array}{ccc} {M_{x}}_{t} & {M_{y}}_{t} & {M_{z}}_{t} \end{array}\right]}^{T}$; $k_{xx}$, $k_{yy}$, and $k_{zz}$ are scale factor coefficients; $k_{xy}$, $k_{yz}$, and $k_{zx}$ are the transverse coefficients caused by the magnetometer’s axes non-orthogonality; $b_{m_{x}}$, $b_{m_{y}}$, and $b_{m_{z}}$ are correction coefficients for biases created by local magnetic field.

According to Figure 1, the observation model of the magnetometer is

(40) $$M_{M}=R_{MB}R_{WB}^{T}H_{W}$$

where $R_{MB}$ is the relative rotation matrix from the body frame $f_{B}$ to the magnetometer’s frame $f_{M}$ and is determined though calibration.

Following from the magnetometer’s observation model, the Jacobians of the measurement function with respect to the variable to optimize $R_{WB}$ are

(41) $$J_{\frac{M}{R}}=\frac{\partial M_{M}}{\partial R_{WB}}=R_{MB}R_{WB}^{T}H_{W}^{\times }$$

where the perturbation is defined on the right side of $R_{WB}$ (globally defined).

## 5. Overall System Structure

**Figure 2:**
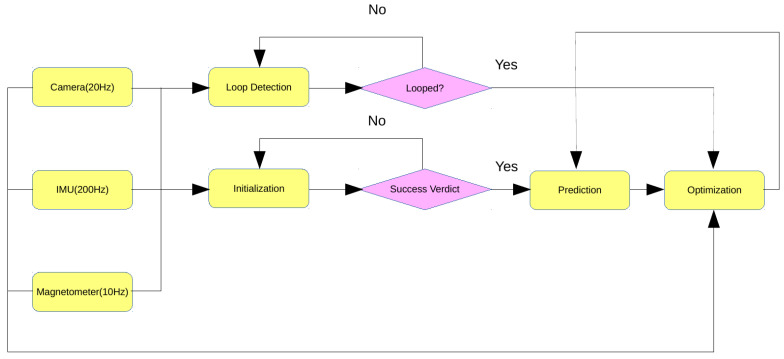
Overall system structure.

Figure 2 depicts the system’s structure plainly.

## 6. Visual-Inertial-Magnetic Initialization

The initialization of monocular visual inertial odometry is as crucial as it is intricate, on account of its precarious structure. Futile or incomplete initialization spell trouble for the entire system. On the one hand, monocular vision calls for a certain length of translation long enough to reflect the depths of key points, on the other hand, the projection of gravity onto the body frame can only be calculated when there’s no extra acceleration other than gravity.

**Figure 3:**
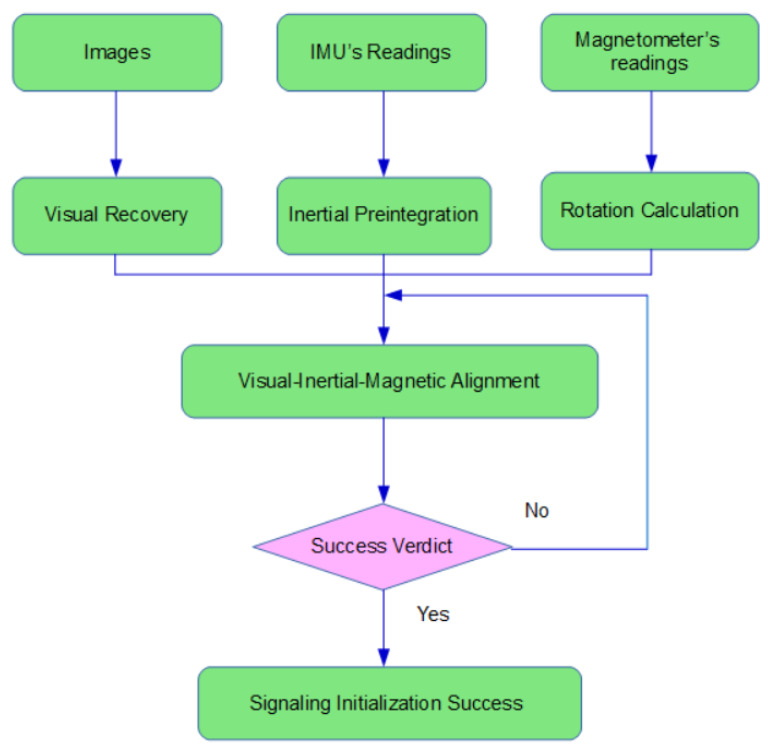
The flowchart of initialization.

This section intends to present and lay out a novel and efficacious procedure of initialization. As drawn out in Section 2, successful initialization is a prerequisite for the system to start off properly. How accurate the initial parameters are estimated will play a huge part in how stably and smoothly the system operates. The involvement of magnetometers introduces extra parameters to be determined, namely the initial magnetic bearing. The proposed method delivers visual-inertial-magnetic initialization with efficacy and credibility assured in some measure by deciding whether or not it is initialized successfully through a set of specific criteria. Figure 3 illustrates the procedure of initialization.

As opposed to VI-SLAM, the system uses magnetic information as rotation constraint during visual recovery with the intention of expediting the initialization process and enhancing its precision.

### 6.1. Visual Recovery and Rotation Calculation through Magnetism

The task the visual module undertakes is estimating the relative transformation with respect to the first frame by vision itself. Using a monocular camera without depth information indicates the first step is to extract from two selected images the essential or homography matrix which can be decomposed to recover the transformation between the images. The two images for recovering poses are selected if there’s enough parallax between them. The essential matrix is better at computing poses if the camera’s moved, whereas if it is only rotated without translation the homography matrix fare better. The problem is there’s no way of establishing whether or not the camera’s moved because either pure rotation or translation causes parallax between two images. For better robustness, we compute and decompose both of them and check through whose transformation the reprojection is smaller to decide to use which matrix. Key points tracked in the two images are triangulated to determine depths if there’s sufficient translation between them. Those 3D key points are then utilized to execute PnP (Perspective-n-Point) on all the intervening key frames under the auspices of BA (Bundle Adjustment).

Magnetic information is also exploited for rotation estimation by incorporating its measurements into the BA problem as extra cost functions in Equation (42).

(42) $$E_{M}=M_{M_{k+1}}-R_{WB_{k+1}}^{T}R_{WB_{k}}R_{BM}M_{M_{k}}$$

where $M_{k}$ denotes magnetometer’s *k*th frame and $R_{BM}$ is the rotation from magnetometer’s frame to body’s frame.

### 6.2. Visual-Inertial-Magnetic Alignment

Before alignment, gyroscope biases need to be worked out. The reason why only gyroscope biases are computed is because attitude holds much more influence on pose estimation as its estimates dictate whether gravity can be rightly projected onto the body frame. By solving Equation (43) through least squares, gyroscope biases $b_{w_{B_{k}}}$ can be obtained:

(43) $$\Delta R_{B_{k}B_{k+1}}R\left(\frac{\partial \Delta R_{B_{k}B_{k+1}}}{\partial b_{w_{B_{k}}}}b_{w_{B_{k}}}\right)=R_{WC_{k}}^{T}R_{BC}^{T}R_{WC_{k+1}}R_{BC}^{T}$$

where $\Delta R_{B_{k}B_{k+1}}$ is the rotational preintegration and $\frac{\partial \Delta R_{B_{k}B_{k+1}}}{\partial b_{w_{B_{k}}}}$ is its partial derivative with respect to gyroscope biases. Section 4.2 lays out how to maintain this partial derivative.

(44) $$\Delta P^{B_{k}B_{k+1}}=R_{WB_{k}}^{T}sR_{C}^{B}P_{W}^{WC_{k+1}}-R_{WB_{k}}^{T}\left(sR_{C}^{B}P_{W}^{WC_{k}}+\Delta tV_{W}^{WB_{k}}-\frac{1}{2}\Delta t^{2}G_{W}\right)$$

(45) $$\Delta V^{B_{k}B_{k+1}}=R_{WB_{k}}^{T}V_{W}^{WB_{k+1}}-R_{WB_{k}}^{T}\left(V_{W}^{WB_{k}}-\Delta tG_{W}\right)$$

where s, $V_{W}^{WB_{k}}$, $V_{W}^{WB_{k+1}}$, and $G_{W}$ are the scalar factor, velocity, and the vector of gravity to be determined. Obviously, $P_{W}^{WC_{k}}$ and $P_{W}^{WC_{k+1}}$ come from the visual recovery module. Every combination of adjacent images and the preintegration in-between forms a set of equations like (44), stacking up into a least squares problem.

The process described above is merely visual-inertial alignment after which the *z*-axis of the world frame is aligned with the vector of gravity, velocity states projected onto the world frame, and key points’ depths scaled to proper size. The next step is to align the magnetometer with the world frame by making its projection on the xy plane of the world frame parallel with the *x*-axis.

After the complete alignment, the world frame’s *z*-axis is parallel with the direction of gravity and *x*-axis with magnetic north.

### 6.3. Initialization Completion Verdict

It is not beyond the bounds of possibility that parameters initialized could be dubious. A common case explaining this phenomenon is when the system keeps moving in one direction at a constant speed, not administering sufficient excitation to IMU.

Inspired by [32,33], we examines initialization’s efficacy by reviewing estimation error that links in to a degree with the uncertainty of the initialization system.

With the estimation error satisfactorily low, the system will be notified and go to the optimization stage.

## 7. Joint Nonlinear Optimization

As regards calculation of Jacobians, it warrants mention that the perturbation is defined on the right side where rotation variables are involved, as evidenced in [34] to have better properties.

(46) $$T\leftarrow \delta T⊗T\left\{\begin{array}{c} R\leftarrow \delta R\cdot R\\ P\leftarrow P+\delta P \end{array}\right.$$

Different definitions of the perturbation for differentiation certainly lead to different Jacobians and inconsistency of which side the perturbation is operated on and how the Jacobians are calculated is heading for failure in the process of optimization.

### 7.1. Visual Constraint

Feature points for tracking are extracted from every image [35] and data association across image frames is realized by tracking them through optical flow [36], which, as exhibited in real-time operation, relieves the visual front-end of heavy computation because it does not have to describe features and subsequently match them as do standard feature-based methods. The number of successfully tracked points will surely diminish as images arrive and pass frame after frame either because of points moving out of the image region or simply of tracking failure. Additional features are extracted in every frame where the number of successfully tracked points drops to a certain amount. Non-maximum suppression is applied to the extraction of key points to obtain a more even distribution of points that would otherwise cluster around a few areas little more than single features.

As key points have to be dispersed to better represent the whole image, so do frames need to be selected as key frames and the number of them be curtailed to avoid redundancy. Other than representing a solitary frame and indicating a static state through small errors of re-projection, frames with little parallax in-between or even identical due to a stationary state hold little significance for the whole, probably quite long and large, trajectory and map retained in the system for loop closure detection and other uses. A key frame ought to hold enough connections with its previous and next key frames through co-visibility [37] for pose coverage and efficient estimation in regard to the local graph while being distinct enough from its adjacent ones for the conciseness of the whole pose-graph. In our formulation, whether or not a frame is “key” is conditioned by its key points’ association with the previous and next frames, or specifically, the ratio of the number in the next frame to that in the current frame of successfully tracked key-points that originate from the previous or older frames. A high value (say 0.8) of this ratio suggests that the majority of key points tracked from previous frames to the current one are well observed again in the next frame and thus this frame may be considered to be redundant in the presence of its neighbours, whereas the lower the ratio is, the more contributory the frame is to the observation and retention of landmarks. A practice is to pre-set a ratio threshold above which a frame is deemed ‘non-key’ and is consequently going to be marginalized out after the current round of optimization.

We use a plain pinhole camera as the visual sensing module with an ordinary projection model that has been presented in Section 4.1. The re-projection error is

(47) $$E\left(i,k\right)=Z_{k}^{i}-\pi \left({T_{\mathrm{BC}}}^{T}{T_{\mathrm{WB}}}^{T}{L_{W}^{\mathrm{WL}}}^{i}\right)$$

where *i* indexes landmarks and *k* denotes frames, variables to be optimized in bold type. π(.) is the projection function whose Jacobian matrix ($J_{pro_{2D\to 3D}}$) with respect to the original 3D point vector has been demonstrated in Section 4.1. $T_{BC}$ is the transformation from $f_{C}$ to $f_{B}$ that is regarded as being constant provided the sensors are rigidly fixed on the platform, and the optimization of which is thus optional with it calibrated in advance.

The Jacobians of the re-projection error with respect to each variable are as follows:

(48) $$\frac{\partial E(i,k)}{\partial R_{BC}}=-J_{pro_{2D\to 3D}}\cdot R_{BC}^{T}{\left(T_{WB}^{T}{L_{W}^{WL}}^{i}-t_{BC}\right)}^{\times }$$

(49) $$\frac{\partial E(i,k)}{\partial t_{BC}}=J_{pro_{2D\to 3D}}\cdot R_{BC}^{T}$$

(50) $$\frac{\partial E(i,k)}{\partial R_{WB}}=-J_{pro_{2D\to 3D}}\cdot R_{BC}^{T}R_{WB}^{T}{\left({L_{W}^{WL}}^{i}-t_{WB}\right)}^{\times }$$

(51) $$\frac{\partial E(i,k)}{\partial t_{WB}}=J_{pro_{2D\to 3D}}\cdot R_{BC}^{T}R_{WB}^{T}$$

(52) $$\frac{\partial E(i,k)}{\partial {L_{W}^{WL}}^{i}}=-J_{pro_{2D\to 3D}}\cdot T_{BC}^{T}T_{WB}^{T}$$

where $t_{BC}$ and $t_{WB}$ are the translation parts of $T_{BC}$ and $T_{WB}$. $J_{pro_{2D\to 3D}}$ are the Jacobian matrices of pixel coordinates with respect to normalized 3D points as duduced in Section 4.1. The above jacobians are derived with the rotation and translation variables treated separately as they are when being updated, rather than computed as a whole as in [38].

### 7.2. Inertial Constraint

As inertial observations take the form of the integration of measurements between two adjacent frames aligned in time with camera frames, they somewhat resemble a measurement of the relative transformation between each two frames, save that gravity is incorporated in the pre-integration. The equations below express the observation model of pre-integration.

(53) $$\begin{array}{cc} \Delta P^{B_{k}B_{k+1}} & ={R_{WB_{k}}}^{T}P_{W}^{WB_{k+1}}-{R_{WB_{k}}}^{T}\left(P_{W}^{WB_{k}}+\Delta tV_{W}^{WB_{k}}-\frac{1}{2}\Delta t^{2}G_{W}\right) \end{array}$$

(54) $$\begin{array}{cc} \Delta V^{B_{k}B_{k+1}} & ={R_{WB_{k}}}^{T}V_{W}^{WB_{k+1}}-{R_{WB_{k}}}^{T}\left(V_{W}^{WB_{k}}-\Delta tG_{W}\right) \end{array}$$

(55) $$\begin{array}{cc} \Delta R_{B_{k}B_{k+1}} & ={R_{WB_{k}}}^{T}R_{WB_{k+1}} \end{array}$$

where ${\left(.\right)}^{B_{k}}$ and ${\left(.\right)}^{B_{k+1}}$ denote the first and last frames of the pre-integration and the variables to optimize are highlighted in bold type.

The whole state vector to be optimized according to the pre-integration observation from $f_{B_{k}}$ to $f_{B_{k+1}}$ is given below:

(56) $$\begin{array}{c} \left[\begin{array}{cccccccccc} P_{W}^{WB_{k}} & V_{W}^{WB_{k}} & \theta_{WB_{k}} & b_{w_{B_{k}}} & b_{a_{B_{k}}}\; & P_{W}^{WB_{k+1}} & V_{W}^{WB_{k+1}} & \theta_{WB_{k+1}} & b_{w_{B_{k+1}}} & b_{a_{B_{k+1}}} \end{array}\right] \end{array}$$

with the corresponding residuals:

(57) $$\begin{array}{c} \left[\begin{array}{ccccc} R_{B_{k}B_{k+1}}\left(P\right) & R_{B_{k}B_{k+1}}\left(V\right) & R_{B_{k}B_{k+1}}\left(\theta \right) & R_{B_{k}B_{k+1}}\left(b_{w}\right) & R_{B_{k}B_{k+1}}\left(b_{a}\right) \end{array}\right] \end{array}$$

(58) $$\begin{array}{c} R_{inertia}=\left[\begin{array}{c} R_{B_{k}B_{k+1}}\left(P\right)\\ R_{B_{k}B_{k+1}}\left(V\right)\\ R_{B_{k}B_{k+1}}\left(\theta \right)\\ R_{B_{k}B_{k+1}}\left(b_{w}\right)\\ R_{B_{k}B_{k+1}}\left(a_{w}\right) \end{array}\right]=\left[\begin{array}{c} \Delta P^{B_{k}B_{k+1}}-{R_{WB_{k}}}^{T}\left(P_{W}^{WB_{k+1}}-P_{W}^{WB_{k}}-\Delta tV_{W}^{WB_{k}}+\frac{1}{2}\Delta t^{2}G_{W}\right)\\ \Delta V^{B_{k}B_{k+1}}-{R_{WB_{k}}}^{T}\left(V_{W}^{WB_{k+1}}-V_{W}^{WB_{k}}+\Delta tG_{W}\right)\\ \Delta R_{B_{k}B_{k+1}}^{T}⊕{R_{WB_{k}}}^{T}R_{WB_{k+1}}\\ b_{w_{B_{k+1}}}-b_{w_{B_{k}}}\\ b_{a_{B_{k+1}}}-b_{a_{B_{k}}} \end{array}\right] \end{array}$$

where the variables to be optimized are in bold type as before. Note that the representations of $R_{B_{k}B_{k+1}}\left(b_{w}\right)$ and $R_{B_{k}B_{k+1}}\left(a_{w}\right)$ indicate that by nature the two biases would not change over a period of pre-integration.

The optimization of the variables for inertial residuals needs Jacobians, as do other optimization problems, around various linearized points to nudge variables towards at least a suboptimal solution. While the analytical form of Jacobians may not necessarily be required as there are available optimization modules capable of automatic differentiation, the analytical form is favourable nonetheless in its optimality and efficiency. The equations below present the Jacobians of pre-integration residuals with respect to variables

- Jacobians of $R_{B_{k}B_{k+1}}\left(P\right)$
  (59) $$\begin{array}{cc} \frac{\partial R(P)}{\partial P_{W}^{WB_{k}}} & =R_{B_{k}W} \end{array}$$
  (60) $$\begin{array}{cc} \frac{\partial R(P)}{\partial V_{W}^{WB_{k}}} & =R_{B_{k}W}\Delta t \end{array}$$
  (61) $$\begin{array}{cc} \frac{\partial R(P)}{\partial P_{W}^{WB_{k+1}}} & =-R_{B_{k}W} \end{array}$$
  (62) $$\begin{array}{cc} \frac{\partial R(P)}{\partial R_{B_{k}W}} & =-R_{B_{k}W}{\left(P_{W}^{WB_{k+1}}-P_{W}^{WB_{k}}-\Delta tV_{W}^{WB_{k}}+\frac{1}{2}\Delta t^{2}G_{W}\right)}^{\times } \end{array}$$
  (63) $$\begin{array}{cc} \frac{\partial R(P)}{\partial b_{w_{B_{k}}}} & =\frac{\partial \Delta P^{B_{k}B_{k+1}}}{\partial b_{w_{B_{k}}}} \end{array}$$
  (64) $$\begin{array}{cc} \frac{\partial R(P)}{\partial b_{a_{B_{k}}}} & =\frac{\partial \Delta P^{B_{k}B_{k+1}}}{\partial b_{a_{B_{k}}}} \end{array}$$
- Jacobians of $R_{B_{k}B_{k+1}}\left(V\right)$
  (65) $$\begin{array}{cc} \frac{\partial R(V)}{\partial V_{W}^{WB_{k}}} & =R_{B_{k}W} \end{array}$$
  (66) $$\begin{array}{cc} \frac{\partial R(V)}{\partial V_{W}^{WB_{k+1}}} & =-R_{B_{k}W} \end{array}$$
  (67) $$\begin{array}{cc} \frac{\partial R(V)}{\partial R_{B_{k}W}} & =-R_{B_{k}W}{\left(V_{W}^{WB_{k+1}}-V_{W}^{WB_{k}}+\Delta tG_{W}\right)}^{\times } \end{array}$$
  (68) $$\begin{array}{cc} \frac{\partial R(V)}{\partial b_{w_{B_{k}}}} & =\frac{\partial \Delta V^{B_{k}B_{k+1}}}{b_{w_{B_{k}}}} \end{array}$$
  (69) $$\begin{array}{cc} \frac{\partial R(V)}{\partial b_{a_{B_{k}}}} & =\frac{\partial \Delta V^{B_{k}B_{k+1}}}{b_{a_{B_{k}}}} \end{array}$$
- Jacobians of $R_{B_{k}B_{k+1}}\left(\theta \right)$
  (70) $$\begin{array}{cc} \frac{\partial R(\theta )}{\partial R_{WB_{k}}} & =-\Delta R_{B_{k}B_{k+1}}^{T}\cdot R_{B_{k}W} \end{array}$$
  (71) $$\begin{array}{cc} \frac{\partial R(\theta )}{\partial R_{WB_{k+1}}} & =\Delta R_{B_{k}B_{k+1}}^{T}\cdot R_{B_{k}W} \end{array}$$
  (72) $$\begin{array}{cc} \frac{\partial R(\theta )}{\partial b_{w_{B_{k}}}} & =\frac{\partial \Delta R_{B_{k}B_{k+1}}}{b_{w_{B_{k}}}} \end{array}$$
  (73) $$\begin{array}{cc} \frac{\partial R(\theta )}{\partial b_{a_{B_{k}}}} & =\frac{\partial \Delta R_{B_{k}B_{k+1}}}{b_{a_{B_{k}}}} \end{array}$$
- Jacobians of $R_{B_{k}B_{k+1}}\left(b_{w}\right)$ and $R_{B_{k}B_{k+1}}\left(b_{a}\right)$
  (74) $$\frac{\partial R(b_{w})}{\partial b_{w_{B_{k}}}}=-I\;\frac{\partial R(b_{a})}{\partial b_{a_{B_{k}}}}=-I\;\frac{\partial R(b_{w})}{\partial b_{w_{B_{k+1}}}}=I\;\frac{\partial R(b_{a})}{\partial b_{a_{B_{k+1}}}}=I$$

### 7.3. Magnetic Constraint

Magnetic observation, as laid out in Section 4.3, is the projection of the vector of the EMF onto the body frame:

(75) $$M_{M}=R_{MB}R_{WB}^{T}H_{W}$$

where $R_{MB}$ is the relative rotation matrix from the body frame $f_{B}$ to the magnetometer’s frame $f_{M}$ and is determined though calibration.

With the observation model, it is fairly straightforward to establish the magnetic residual:

(76) $$R\left(M\right)=M_{M}-R_{\mathrm{MB}}{R_{\mathrm{WB}}}^{T}H_{W}$$

where $R_{\mathrm{MB}}$ and $R_{\mathrm{WB}}$ are to be optimized.

The corresponding Jacobians:

(77) $$\frac{\partial R(M)}{\partial R_{\mathrm{MB}}}={\left(R_{MB}R_{WB}^{T}H_{W}\right)}^{\times }\frac{\partial R(M)}{\partial R_{\mathrm{WB}}}=-R_{MB}R_{WB}^{T}H_{W}^{\times }$$

Note that $M_{M}$ and $H_{W}$ are always normalized with norm equal to 1. $H_{W}$ is the vector of the total intensity of the EMF whose projection on the X-Y plane is in the direction of magnetic north cross true north at an angle called magnetic declination which is not concerned in our system since we use magnetometers only for suppressing yaw estimation shift.

### 7.4. Loop Closure Constraint

Loop closure constraints come from the front-end’s detection of loop closures when the robot arrives where it has roamed and thus its trajectory loops. Loop closure constraints take the form of

(78) $$E_{loop}\left(i,j\right)=Z_{j}-\pi \left({{T_{\mathrm{BC}}}_{j}}^{T}{{T_{\mathrm{WB}}}_{j}}^{T}{L_{W}^{WL}}^{i}\right)$$

where *i* and *j* are the indexes of frames between which a loop closure occurs. $f_{i}$ is the older one whose pose together with the landmarks it holds are fixed on the grounds that the older a frame is the less its estimate has drifted. Equation (78) is almost identical to the visual constraint, save that the landmarks of the older frame are in regular type, signaling that they are not to be optimized since their would not be any other variable more precise than the start of the loop.

Apparently, only frames involved in loop closures are to be adjusted, leaving the rest unchanged, if optimization is executed merely on loop closures. That brings about inconsistency in the pose graph. One putative way is to combine odometry constraints and loop closures. Odometry constraints are relative pose transformations obtained from the current pose graph.

(79) $$T_{ii+1}=T_{wi}^{-1}\cdot T_{wi+1}$$

where $T_{ij}$ is a relative transformation taken from the existing pose graph as a measurement. The standard pose graph optimization problem is then presented as

(80) $$E_{pose\;graph}=\sum_{i,j}E_{loopclosure}\left(i,j\right)+\sum_{i}E_{odometry}\left(i,i+1\right)$$

As [39] suggests, optimization of a pose graph is no more than a plain nonlinear least squares problem. What makes it call for extra cautious treatment is its susceptibility to false positive loop closures. Even a single outlier made by the front-end has the potential to bring the optimization into complete divergence or make the pose graph wrongly deform. One way to tackle this is to obviate outliers as early as in the detection stage. By applying RANSAC or other similar strategies under geometric models, outliers can be fairly picked out and eliminated, making false loops less likely to be carried over into the following optimization. Another relies on what is called the robust cost function which bears much resemblance to the Huber function [40] and can mitigate the impact of outliers by downgrading the cost functions to linear functions rather than quadratic ones, where the overall cost is high enough to be deemed harhouring false loops. Neither of these is foolproof. Systems using these methods are liable to suffer from wrongly detected closures all the same. False positive loop constraints are as much problematic as they are difficult to combat. Erroneous edges in the pose graph following detection of false loops can either lead the optimization to diverge or converge to a entirely egregious solution.

Inspired by [39], we adopt the idea that the topology of the pose graph is subject to the optimization with outliers being identified and removed automatically. To actualize this idea, a weighting factor is put on every loop closure constraint as a way of distinguishing normal loop closures from erroneous ones.

(81) $$E_{pose\;graph}=\sum_{i,j}w_{ij}\cdot E_{loopclosure}\left(i,j\right)+\sum_{i}E_{odometry}\left(i,i+1\right)$$

In the above equation, $w_{ij}$ is a weighting factor ranging from 0 to 1 corresponding to whether the constraint is active or deactivated, or rather, removed. As the weighting factor holds influence on the cost value, it indeed makes the topology itself subject to the optimization. The weighting factor is then given by a sigmoid function:

(82) $$w_{ij}=sig\left(s_{ij}\right)=\frac{1}{1+e^{-s_{ij}}}$$

where $s_{ij}$ is a switch variable switches on and off a loop closure constraint during the optimization process, hence the name switch variable. These switch variables are to be optimized together with others and are set to 10 initially, meaning all loop closure constraints are initially activated.

Switch functions alone are apparently not enough to attain what is intended. The optimization module will simply drive all switch function costs to nearly 0 as a result of attempting to minimizing the overall cost. Introducing a penalty cost for each switch variables could counter this phenomenon. Penalty costs are to keep switch variables to the initial value and in the form of prior constraints:

(83) $$\left|\right|\xi_{ij}-s_{ij}{\left|\right|}_{\Xi }$$

where Ξ, the covariance matrix for the penalty cost, is empirically set to $20^{2}$.

Switch functions and prior constraints together enable the optimization unit to winnow out false loops, and the principle behind it is to tap into the knowledge that there’s a certain amount of inconsistency between false and true loops and between false ones themselves in all likelihood while true loops tend to be in agreement with each other, and so what the proposed mechanism does essentially is to always slash the impact of loop closures that are incongruous with other loops.

## 8. Implementation Details and Experiments

### 8.1. Implementation Details

The navigation system’s software is programmed in C++ with recourse to ceres-solver on ROS (Robot Operating System).

The system runs on a standard central processing unit (Intel${}^{®}$ NUC Kit NUC7i7BNH, Intel${}^{®}$ Core™ i7-7567U Processor, 16 GB RAM). The suite of sensors is rigidly installed on top of the vehicle and collects data as it is moving.

**Figure 4:**
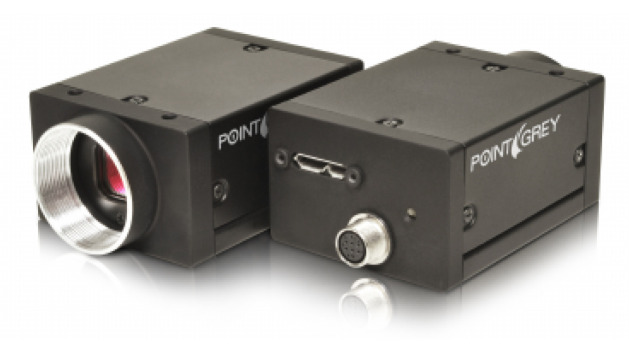
The monocular camera (Point Grey Grasshopper3) used. It outputs grey images of 960×600 at 20 Hz through USB 3.0.

**Figure 5:**
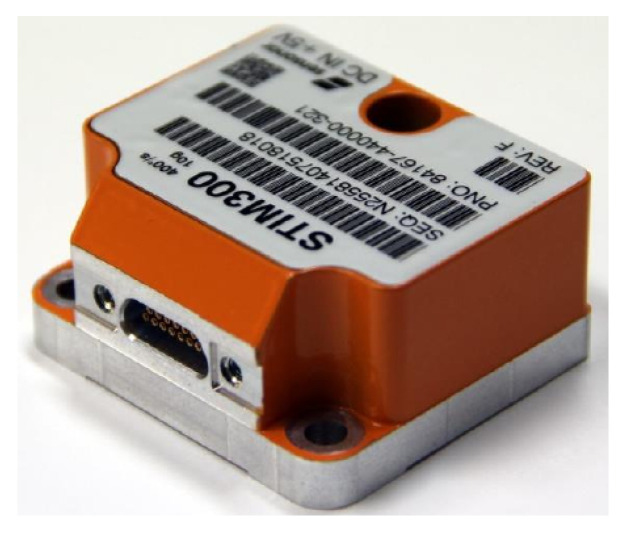
The IMU (STIM300) used that outputs instant angular velocity and acceleration at 200 Hz.

**Figure 6:**
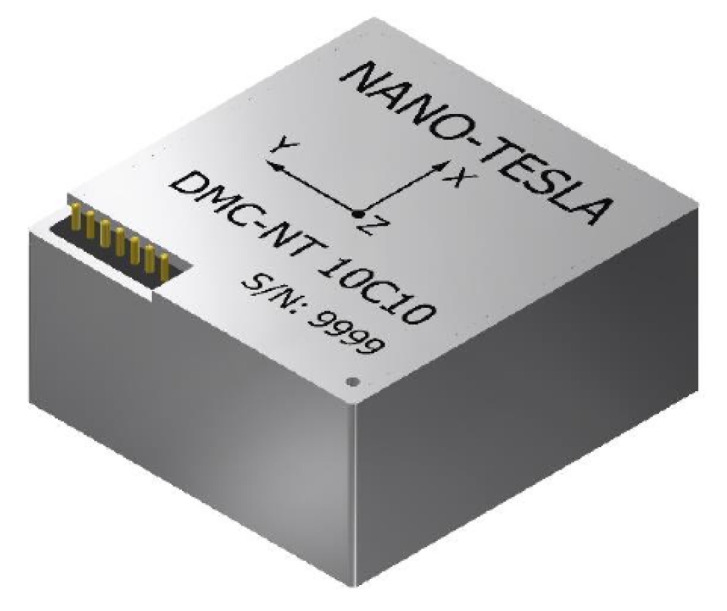
The magnetometer (DMC-NT 10C10) used. The system requests from it the vector of magnetic field 10 times a second.

Figure 4, Figure 5 and Figure 6 are pictures of the sensors employed in the system.

**Figure 7:**
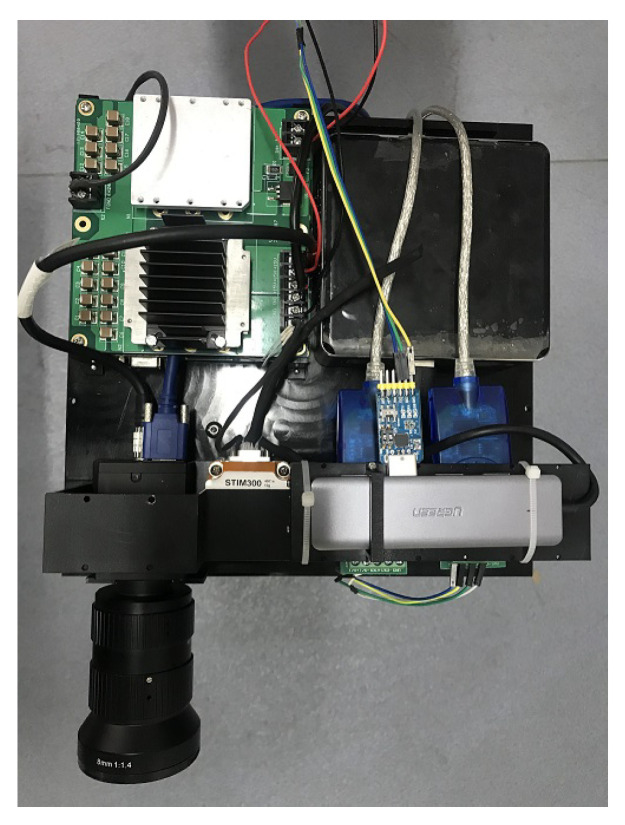
The device we use for data acquisition. The camera and the inertial measurement unit (IMU) are hardware-synchronized with the latter triggering the former through a synchronization signal. The central processing unit is Intel${}^{®}$ NUC(Next Unit of Computing).

Figure 7 is the sensor suite with the CPU.

**Figure 8:**
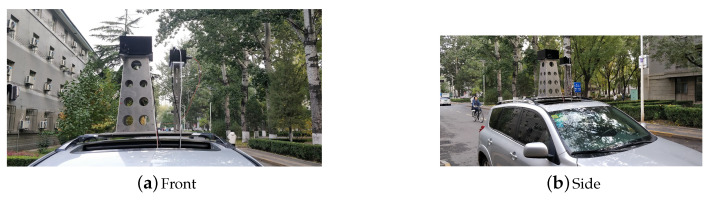
The vehicle used for carrying sensors. The sensors are raised up high away from the top of the vehicle with a stand made of aluminium lest the magnetometer’s measurements be corrupted.

Figure 8 is pictures of the vehicle used.

### 8.2. Experiments

We conducted three large-scale outdoor experiments each of which covers over 1 kilometer by collecting and saving data into a bag file for later analysis. We examined the performance of VI-SLAM (visual-inertial SLAM) and VIMO (the proposed visual-inertial-magnetic navigation system) by running them on recorded datasets to glean quantitative results.

**Figure 9:**
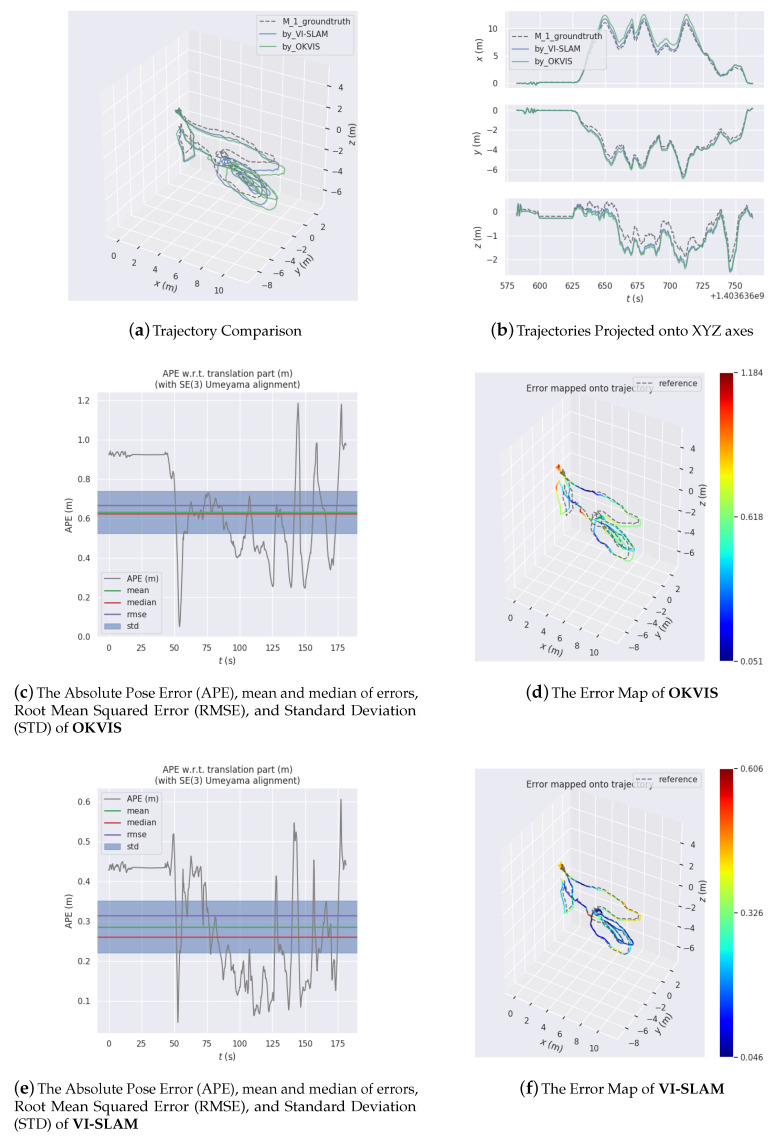
Experiment results on EUROC dataset MH 01 easy.

**Figure 10:**
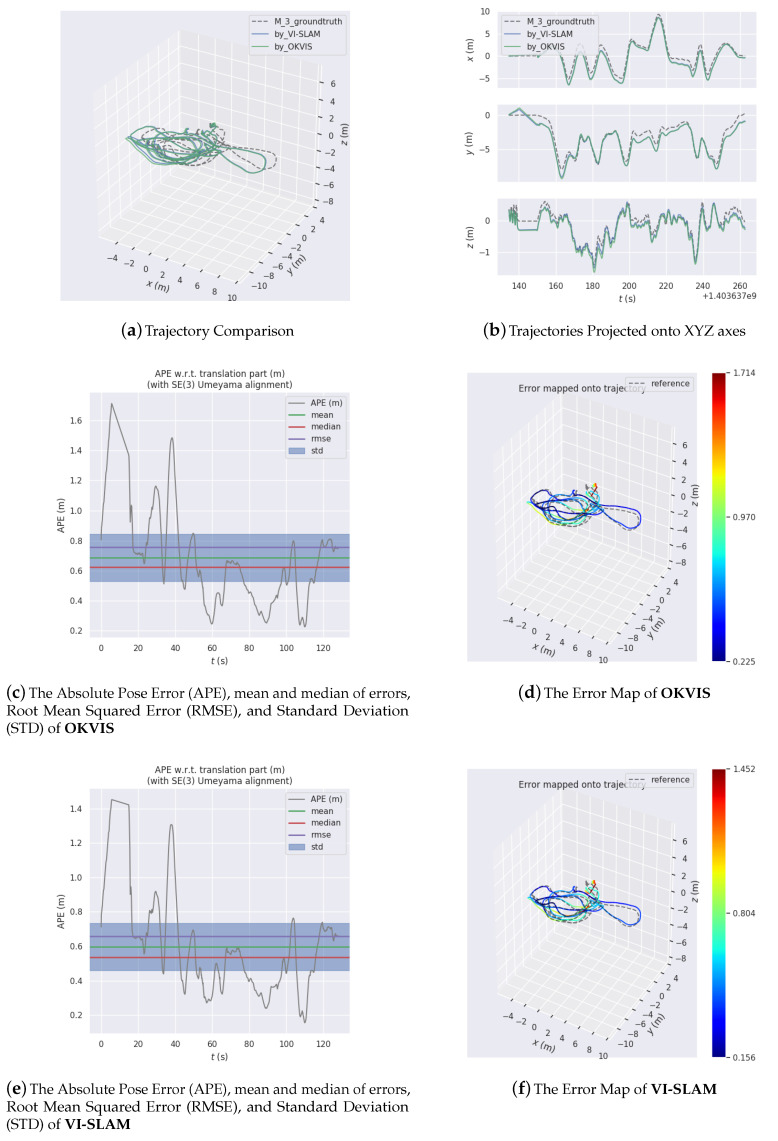
Experiment results on EUROC dataset MH 03 easy.

**Figure 11:**
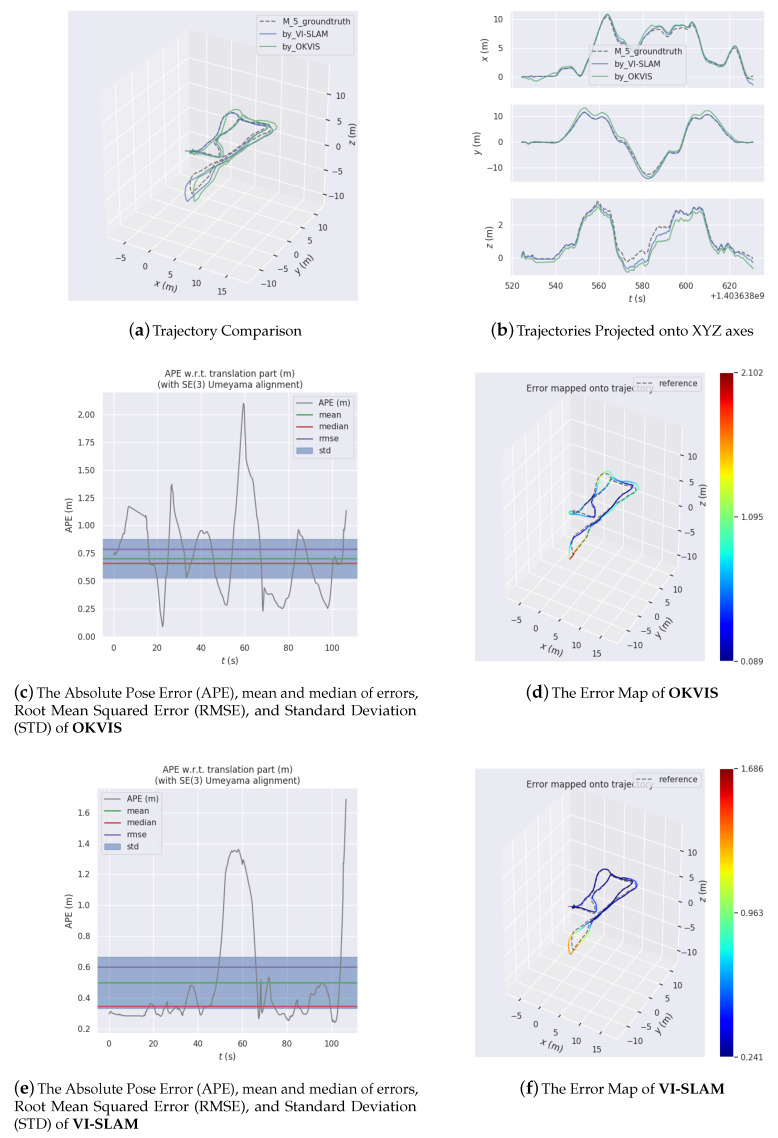
Experiment results on EUROC dataset MH 05 easy.

Figure 9, Figure 10 and Figure 11 are three experiments on EUROC datasets “MH 01 easy”, “MH 03 medium” and “MH 05 difficult” for VI-SLAM and OKVIS. VIMO is not compared with them, since the public datasets do not contain magnetic measurements.

Figure 9, Figure 10 and Figure 11 display estimated trajectories and absolute pose errors by VI-SLAM and OKVIS for comparison.

**Figure 12:**
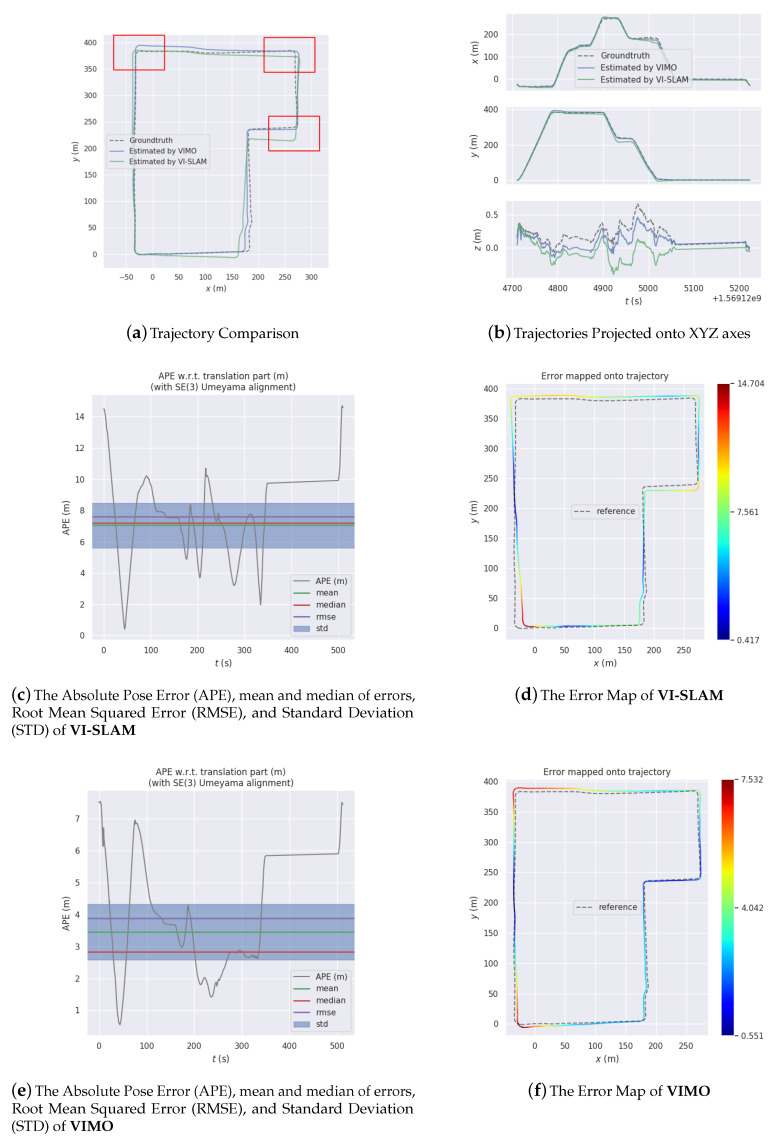
Experiment 1.

**Figure 13:**
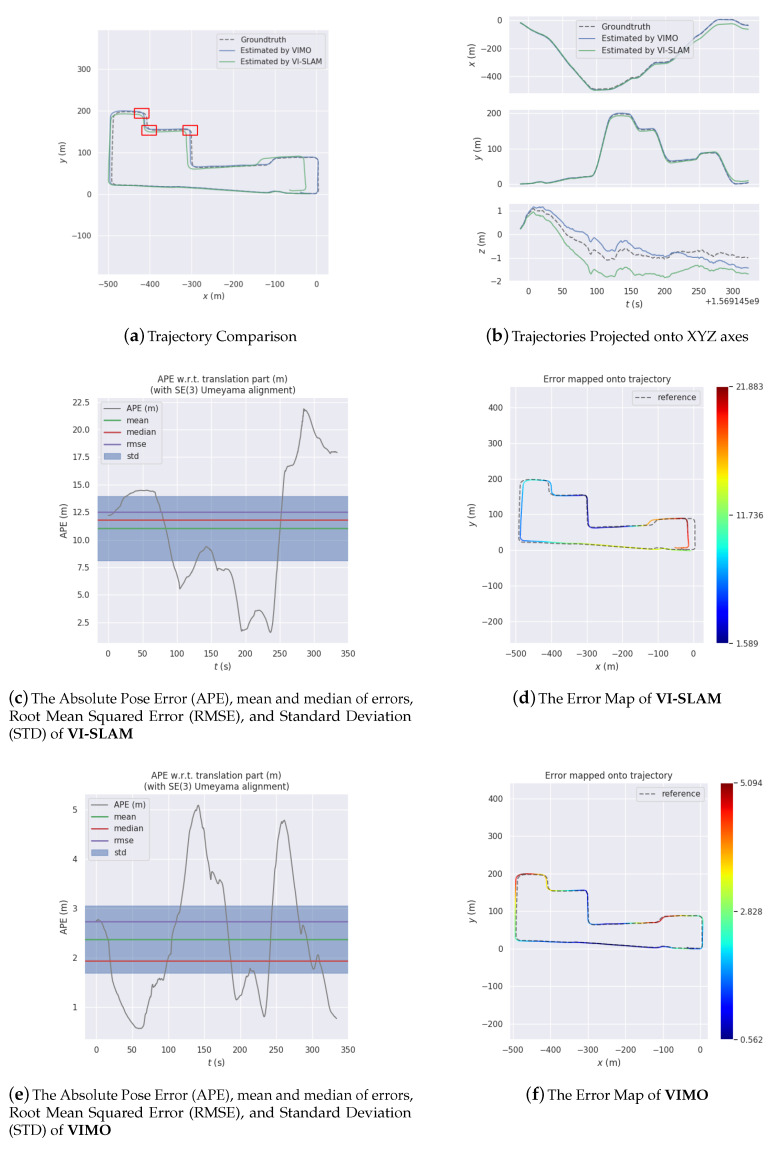
Experiment 2.

**Figure 14:**
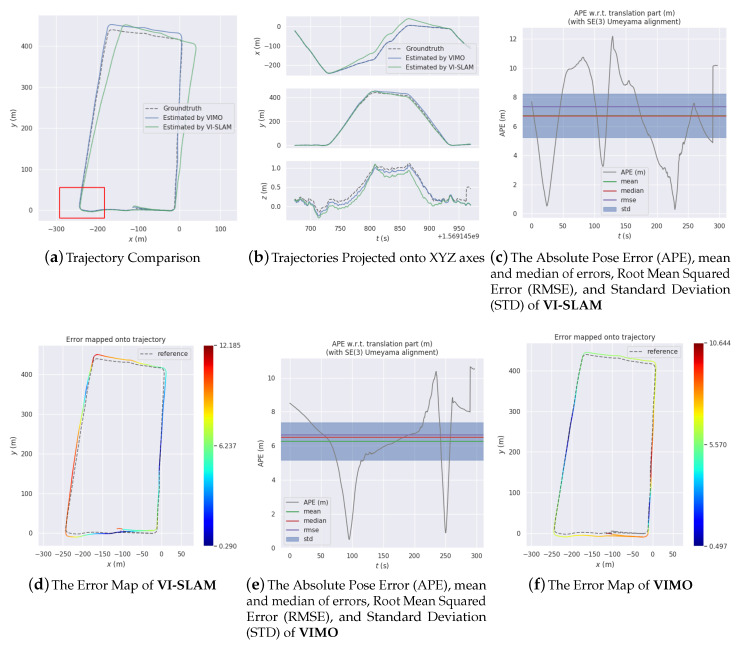
Experiment 3.

Figure 12, Figure 13 and Figure 14 are comparisons of 3 experiments together with error analysis. Manifest improvement shown by VIMO on VI-SLAM in positioning accuracy is validated thought these comparisons.

As is illustrated by the pose error map in Figure 9, the absolute pose error by VI-SLAM is (−0.046 (m) to −0.606 (m)) compared to that by OKVIS (−0.051 (m) to −1.184 (m)).

In Figure 10, for the dataset "MH 03 medium", absolute pose error is dragged down from −1.714 (m) by OKVIS to −1.452 (m) by VI-SLAM.

In Figure 11, since the dataset is labeled ’difficult’, the accuracy has fallen for either system, with −2.102 (m) by OKVIS and −1.686 (m) by VI-SLAM. An absolute decrease of 0.416 (m) in error is shown.

Table 1, Table 2 and Table 3 show differences in accuracy between OKVIS [21] and VI-SLAM for EUROC datasets. It is shown that VI-SLAM generally outperforms OKVIS.

In Table 1, the root square mean error by VI-SLAM (0.314019 m) and that error by OKVIS is (0.623834 m).

In Table 2, all error terms except for root mean square error are smaller by VI-SLAM (Max: 1.45192 m, Mean: 0.597207 m, Median: 0.534837 m, RMSE: 0.656985 m, StD: 0.273813 m ) than by OKVIS (Max: 1.71401 m, Mean: 0.687552 m, Median: 0.623768 m, RMSE: 0.756585 m, StD: 0.315741 m).

In Table 3, VI-SLAM (Max: 1.68566 m, Mean: 0.498092 m, Median: 0.345175 m, RMSE: 0.599893 m, StD: 0.33433 m) outperforms OKVIS (Max: 2.10193 m, Mean:0.704724 m, Median: 0.661593 m, RMSE: 0.786519 m, StD: 0.34925 m) for all error terms.

In Figure 12a, the trajectory estimated by VI-SLAM (Max: 14.7041 m, Mean: 7.0558 m, Median: 7.2115 m, RMSE: 7.6135 m, StD: 2.8604 m) deviates greatly from the groundtruth thanks to a sudden change in the vehicle’s direction, and the error in yawing has since remained, causing it to extend away from the groundtruth, whereas for VIMO (Max: 7.5324 m, Mean: 3.4541 m, Median: 2.8337 m, RMSE: 3.8741 m, StD: 1.7543 m) that issue is so effectively ameliorated that not only the heading estimation is corrected but the overall absolute pose error is better constrained.

In Figure 13a, the contrast becomes even more manifest. VI-SLAM’s trajectory (Max: 21.8827 m, Mean: 11.0507 m, Median: 11.8128 m, RMSE: 12.4916 m, StD: 5.8243 m) goes wildly away from the groundtruth in the wake of the vehicle turning around time after time. By comparison, the efficacy of fusing magnetic information is accentuated. VIMO (Max: 5.0943 m, Mean: 2.3737 m, Median: 1.9370 m, RMSE: 2.7394 m, StD: 1.3673 m) seems impervious to veering, generating much smaller absolute pose error than it would otherwise do.

Figure 14a implies a similar phenomenon. The difference in accuracy between the two begins to build up after the vehicle corners, and the error in VI-SLAM’s estimation (Max: 12.1847 m, Mean: 6.7346 m, Median: 6.7127 m, RMSE: 7.3614 m, StD: 2.9723 m) continues growing larger while VIMO’s (Max: 10.6441 m, Mean: 6.2678 m, Median: 6.5150 m, RMSE: 6.6390 m, StD: 2.1889 m) keeps nearly on groudtruth.

**Figure 15:**
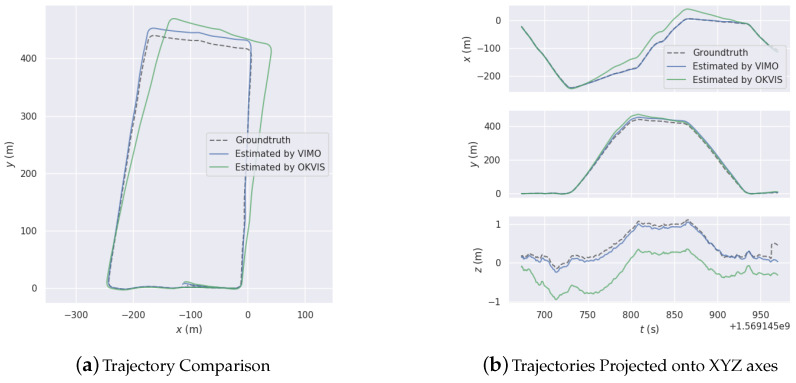

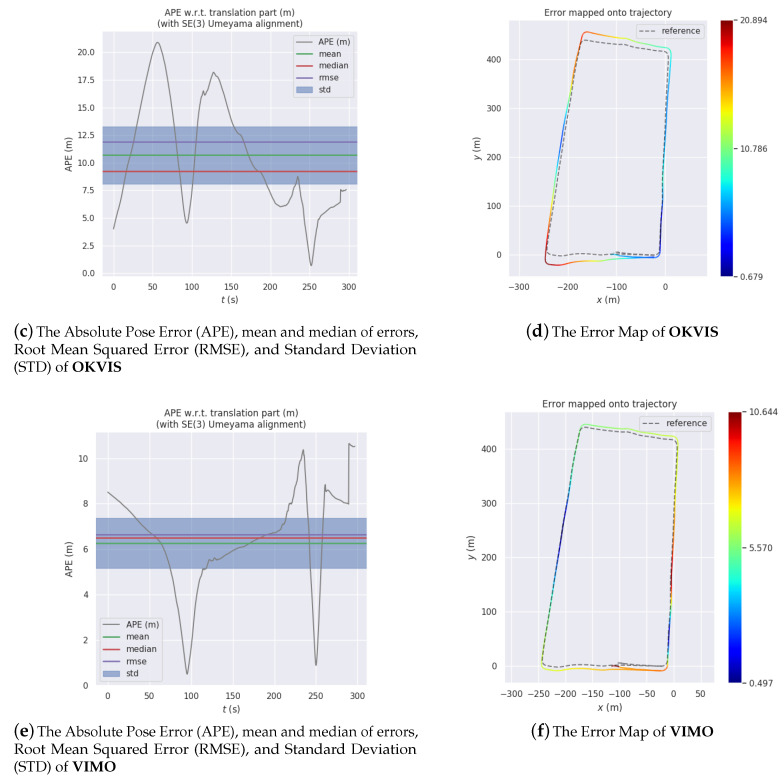
Comparison with OKVIS [21].

Figure 15 compares VIMO with OKVIS [21], a state-of-art method. Improvement in performance is demonstrated.

Another phenomenon warranting consideration is that for VI-SLAM the absolute pose error is larger (Max: 21.8827 m, Mean: 11.0507 m, Median: 11.8128 m, RMSE: 12.4916 m, StD: 5.8243 m) in experiment 2 (Figure 13) than in other experiments, while for VIMO it is quite the contrary. We conjures that it is because the shorter the vehicle travels along a straight line, the lesser error should have been incurred if it were not for accretion in yawing estimation, and since VIMO is immune to yawing deviation it is able to achieve much higher performance, which, again, brings to the fore the significance of magnetic information and the superiority of VIMO.

Various error terms, including max absolute pose error, mean, median, min, root mean squared error, standard deviation of errors, are arranged in Table 4, Table 5 and Table 6. Quantitative comparisons between VI-SLAM and VIMO drawn from the tables reveal the potency of fusing magnetic information into the system.

Table 4, Table 5 and Table 6 shows differences in accuracy among OKVIS [21], VI-SLAM and VIMO. As they demonstrate, both VI-SLAM and VIMO are able to achieve better results than OKVIS do for every error item. In Table 4, the root square mean error for VI-SLAM (7.6135 m) is about half that for OKVIS (16.3864 m), and VIMO makes it even lower (3.8741 m). In Table 5, the difference between VI-SLAM (12.4916 m) and OKVIS (19.9318 m) is not so pronounced as in Table 4, but for VIMO the error is nearly 20 times smaller (2.7394).

## 9. Conclusions

This paper presents a visual-inertial-magnetic navigation system with the originality of exploiting EMF as yaw observation. Respective measurement models are presented in Section 4. Section 5 provides an overview of the system’s structure. We present mathematic fundamentals and theories concerned with visual-inertial-magnetic initialization and non-linear optimization in Section 6 and Section 7, together with other algorithmic details. Lastly, we demonstrate the validity and superiority of our system over visual-inertial-only ones through 3 outdoor large-scale experiments with their error analysis.

According to Section 8.2, VIMO performs localization more accurately than both VI-SLAM and state-of-the-art methods such as OKVIS, slashing errors by half or more (from 16.3864 m to 1.7543 m in Table 4; 19.9318 m to 2.7394 m Table 5; 11.8855 m to 6.6390 m Table 6 in terms of root mean square error).

The most significant implication of the proposed system is that it opens up new vistas for the development of navigation systems with a new combination of sensors and new ways of information fusion and state estimation.

Future research shall follow the line of designing installation mechanisms that can blot out as much magnetic interference as possible, making better use of magnetometers for initialization (for example applying magnetic measurements to the least squares problem in initialization for more accurate estimation), further improving the system’s overall performance.

## Figures and Tables

**Table 1:** Error description of the experiment on EUROC dataset MH 01 easy.

Error Terms (m)	Max	Mean	Median	RMSE	StD
OKVIS [21]	1.1844	0.632254	0.623834	0.666927	0.212242
VI-SLAM	0.606102	0.28583	0.260383	0.314019	0.130034

**Table 2:** Error description of the experiment on EUROC dataset MH 03 medium.

Error Terms (m)	Max	Mean	Median	RMSE	StD
OKVIS [21]	1.71401	0.687552	0.623768	0.756585	0.315741
VI-SLAM	1.45192	0.597207	0.534837	0.656985	0.273813

**Table 3:** Error description of the experiment on EUROC dataset MH 05 difficult.

Error Terms (m)	Max	Mean	Median	RMSE	StD
OKVIS [21]	2.10193	0.704724	0.661593	0.786519	0.34925
VI-SLAM	1.68566	0.498092	0.345175	0.599893	0.33433

**Table 4:** Error description of Experiment 1.

Error Terms (m)	Max	Mean	Median	RMSE	StD
OKVIS [21]	24.7357	12.1936	11.3733	16.3864	5.3875
VI-SLAM	14.7041	7.0558	7.2115	7.6135	2.8604
VIMO	7.5324	3.4541	2.8337	3.8741	1.7543

**Table 5:** Error description of Experiment 2.

Error Terms(m)	Max	Mean	Median	RMSE	StD
OKVIS [21]	26.4495	15.8417	16.7346	19.9318	8.7485
VI-SLAM	21.8827	11.0507	11.8128	12.4916	5.8243
VIMO	5.0943	2.3737	1.9370	2.7394	1.3673

**Table 6:** Error description of Experiment 3.

Error Terms (m)	Max	Mean	Median	RMSE	StD
OKVIS [21]	20.8938	10.6794	9.2471		5.2168
VI-SLAM	12.1847	6.7346	6.7127	7.3614	2.9723
VIMO	10.6441	6.2678	6.5150	6.6390	2.1889

## Abbreviations

The following abbreviations are used in this manuscript:

${\left(.\right)}_{W}$	the World Frame
${\left(.\right)}_{B_{k}}$	the *k*th Body Frame
${\left(.\right)}_{C_{k}}$	the *k*th Camera Frame
${\left(.\right)}_{M_{k}}$	the *k*th Magnetometer Frame
$w_{k}$	the Gyroscope’s Readings
$a_{k}$	the Accelerometer’s Readings
$h_{k}$	the Magnetometer’s Readings
${\left(u,v\right)}_{k}$	the Coordinates of a Feature Point in the *k*th Image
*P*	the Position
*q*	the Body Orientation Quaternion
*V*	the Velocity
$b_{g}$	the Gyroscope’s Biases
$b_{a}$	the Accelerometer’s Biases
$q_{BC}$	the Transformation from Frame *C* to Frame *B*
$L_{W}$	a Landmark Expressed Homogeneously with $L_{W}=\left[\begin{array}{c} x,y,z,1 \end{array}\right]$
$\theta^{\times }$	the Askew Matrix of the Vector $\theta^{\times }$
$J_{r}$	the Right-hand Jacobian
$J_{l}$	the Left-hand Jacobian
$p_{1}$$p_{2}$	the Tangential Distortion
*r*	the Distance of Point from the Origin of the Coordinate system
$n_{w}$	the Gyroscope’s White Noise
$n_{a}$	the Accelerometer’s White Noise
